# Association of English Language Proficiency With Hospitalization Cost, Length of Stay, Disposition Location, and Readmission Following Total Joint Arthroplasty

**DOI:** 10.1001/jamanetworkopen.2022.1842

**Published:** 2022-03-10

**Authors:** Solmaz P. Manuel, Kevin Nguyen, Leah S. Karliner, Derek T. Ward, Alicia Fernandez

**Affiliations:** 1 Department of Anesthesia and Perioperative Care, University of California San Francisco School of Medicine, San Francisco; 2 University of California San Francisco School of Medicine, San Francisco; 3 Department of Medicine, University of California San Francisco School of Medicine, San Francisco; 4 Multiethnic Health Equity Research Center, University of California San Francisco, San Francisco; 5 Department of Orthopedic Surgery, University of California San Francisco School of Medicine, San Francisco; 6 University of California San Francisco Center for Vulnerable Populations, Zuckerberg San Francisco General Hospital, San Francisco

## Abstract

This cohort study examines patient outcomes following total joint arthroplasty to evaluate their association with limited English proficiency.

## Introduction

Total joint arthroplasty (TJA), including hip and knee arthroplasty, is the most common inpatient surgery for Medicare beneficiaries, with an incidence that is expected to increase substantially as the population ages.^1^ To control associated increases in health care costs, surgical bundled payment models have been introduced.^1,2^ This payment reform places pressure on health care facilities to decrease costs, and has increased scrutiny of patient populations at greatest risk for high-cost admissions.^2^

Currently, 22% of adults in the US speak a primary language other than English, and a large proportion of these individuals have limited English proficiency (LEP), which has been independently associated with worse outcomes across multiple health care settings that involve complex communication.^3,4^ Racial and ethnic disparities in outcomes after TJA have been persistent and well documented; however, little is known about the independent association of LEP with perioperative health care and recovery after TJA. In this cohort study we examine the association of LEP with surgical admission length of stay (LOS), discharge destination, hospitalization cost, and rate of 30-day readmission after TJA.

## Methods

This study used electronic health data of patients aged 18 years or older who underwent TJA from 2015 to 2019 at an urban academic medical center (eAppendix in the Supplement). Institutional review board approval was obtained with waiver of informed consent given the minimal risk and use of deidentified data. This study followed the Strengthening the Reporting of Observational Studies in Epidemiology (STROBE) reporting guideline.^5^

Primary outcomes included LOS, total hospitalization cost, discharge disposition, and rate of 30-day hospital readmission. The primary variable was LEP, defined as a language preference other than English and request for interpreter services. Covariates were chosen a priori according to prior literature.

Statistical comparisons between LEP and English-proficient groups were performed using 2-sided χ^2^, Fisher exact, Wilcoxon rank-sum, and *t* tests as appropriate, with *P* < .05 denoting significance. Multivariable models were used to examine the association between LEP and primary outcome variables adjusting for covariates of interest. The distribution of LOS and total hospitalization cost both exhibited overdispersion caused by right skew, which was accommodated using negative binomial regression. Binomial logistic regressions were used to understand the association of LEP with discharge disposition and 30-day readmission rates. Analyses were performed using Stata statistical software version 15.1 (StataCorp). Data were analyzed from May to December 2021.

## Results

Of the 4721 patients included in this study, 2671 (56.6%) were female, mean (SD) age was 64.0 (12.5) years, and 369 (7.8%) had LEP (Table 1). The most common primary languages spoken by patients with LEP were Spanish (140 patients [37.0%]) or a Chinese language (125 patients [33.1%]). Among other differences, patients with LEP were older, more likely to be women, and have a higher American Society of Anesthesiologists status.^6^

**Table 1.** Characteristics of Patients Undergoing Lower Extremity Total Arthroplasty Surgery, by English Proficiency

Characteristic	Patients, No. (%)			*P* value
	Total (N = 4721)	English proficient (n = 4343)	Limited English proficiency (n = 378)	
Surgical procedure, No. of patients				
Hip	2573	2406	167	NA
Knee	2148	1937	211	
Age, mean (SD), y	64.0 (12.5)	63.5 (12.2)	70.1 (14.2)	<.001^a^
Sex				
Male	2049 (43.4)	1935 (44.6)	114 (30.2)	<.001^b^
Female	2672 (56.6)	2408 (55.4)	264 (69.8)	
Race and ethnicity				
Asian or Pacific Islander	454 (9.6)	288 (6.6)	166 (43.9)	<.001^b^
Black	344 (7.3)	343 (7.9)	1 (0.3)	
Hispanic or Latino^c^	420 (8.9)	287 (6.6)	133 (35.2)	
White	3259 (69.0)	3208 (73.9)	51 (13.5)	
Other^d^	244 (5.2)	217 (5.0)	27 (7.1)	
Language spoken				
English	4338 (91.9)	4338 (99.9)	0	<.001^b^
Spanish	140 (3.0)	0	140 (37.0)	
Chinese (multiple languages)	125 (2.6)	0	125 (33.1)	
Other, not English	118 (2.5)	5 (0.1)	113 (29.9)	
Primary insurance type				
Private	1753 (37.1)	1708 (39.3)	45 (11.9)	<.001^b^
Public	663 (14.0)	551 (12.7)	112 (29.6)	
Medicare	2305 (48.8)	2084 (48.0)	221 (58.5)	
American Society of Anesthesiologists status				
1	303 (6.4)	293 (6.7)	10 (2.6)	<.001^e^
2	2947 (62.4)	2727 (62.8)	220 (58.2)	
3	1423 (30.1)	1280 (29.5)	143 (37.8)	
4	48 (1.0)	43 (1.0)	5 (1.3)	
Body mass index, mean (SD)^f^	28.9 (6.1)	29.0 (6.1)	28.1 (5.9)	.01^a^
Case classification				
Elective	386 (8.2)	306 (7.0)	80 (21.2)	<.001^b^
Nonelective	4335 (91.8)	4037 (93.0)	298 (78.8)	
Case length, mean (SD), min	123.8 (55.5)	123.5 (55.1)	127.0 (60.2)	.24^a^
Estimated blood loss, mean (SD), mL	301.3 (357.4)	302.7 (356.4)	284.4 (368.6)	.34^a^

^a^ *P* values were calculated with 2-sample *t* test.

^b^ *P* values were calculated with Pearson χ^2 ^test.

^c^ Hispanic or Latino category includes anyone who self-identified as Hispanic or Latino ethnicity regardless of race.

^d^ Other category includes patients identifying as American Indian or Alaskan Native, as well as those with unknown or unspecified race or ethnicity.

^e^ *P* values were calculated with Fisher exact test.

^f^ Body mass index is calculated as weight in kilograms divided by height in meters squared.

In univariate analysis, patients with LEP who underwent arthroplasty had longer LOS (median [IQR], 3 [2-4] days vs 2 [1-3] days), higher costs of hospitalization (median [IQR] $15 000 [$13 000-$22 000] vs $14 000 [$12 000-$19 000]), and were more likely to be discharged to a skilled care facility (161 patients [42.6%] vs 889 patients [20.5%]) compared with patients with English proficiency. There was no difference in 30-day readmission rates by language status (Table 2).

**Table 2.** Unadjusted and Adjusted Association of Limited English Proficiency With Postoperative Outcomes for Patients Undergoing Lower Extremity Total Joint Arthroplasty

Outcome variables	Univariate analyses^a^				Adjusted multivariable models^b^	
	Total (N = 4721)	English proficient (n = 4343)	Limited English proficiency (n = 378)	*P* value	Adjusted IRR or OR (95% CI)	*P* value
Length of stay, median (IQR), d	2 (1-3)	2 (1-3)	3 (2-4)	<.001^c^	1.15 (1.07-1.25)^d^	<.001
Total costs, median (IQR), $	14 264 (12 184-19 388)	14 179 (12 142-19 207)	15 387 (13 016-21 566)	<.001^c^	1.08 (1.04-1.12)^d^	<.001
Discharge to skilled facility, patients, No. (%)	1050 (22.3)	889 (20.5)	161 (42.6)	<.001^e^	1.41 (1.03-1.93)^f^	.03
30-d Readmission, patients, No. (%)	342 (7.2)	312 (7.2)	30 (7.9)	.59^e^	0.80 (0.49-1.28)^f^	.35

Abbreviations: IRR, incidence rate ratio; OR, odds ratio.

^a^ Univariate data are presented as median (IQR) for continuous measures or as frequency (percentage) for binary measures.

^b^ Negative binomial regression models accommodated right skew exhibited by length of stay and total cost; the resulting IRRs are given. Logistic regression analyses determined ORs for dichotomous outcome variables. IRRs and ORs were adjusted for race and ethnicity, age, sex, primary insurance, American Society of Anesthesiologists status (proxy for medical comorbidities), body mass index, surgical case class, case length, and estimated blood loss. Length of stay and cost rate ratios additionally were adjusted for disposition location. Reference group is patients with English proficiency.

^c^ *P* values were calculated with Wilcoxon rank-sum test.

^d^ Data are IRRs.

^e^ *P* values were calculated with Pearson χ^2 ^test.

^f^ Data are ORs.

In multivariable models adjusted for age, sex, race and ethnicity, body mass index, primary insurance, American Society of Anesthesiologists physical classification status, elective vs urgent case classification, surgical case length, estimated blood loss, and discharge disposition, LEP was associated with longer LOS (incidence rate ratio, 1.15; 95% CI, 1.07-1.25), higher hospitalization costs (incidence rate ratio 1.08; 95% CI, 1.04-1.12), and increased discharge to skilled care (odds ratio, 1.41; 95% CI, 1.03-1.93), but not with 30-day readmission (odds ratio, 0.80; 95% CI, 0.49-1.28) (Table 2).

## Discussion

In this cohort study, we showed that limited English proficiency is independently associated with longer LOS, higher hospitalization costs, and discharge to skilled care after TJA. We acknowledge limitations inherent to observational analyses of electronic health record–derived variables (eAppendix in the Supplement). However, if further research confirms that communication barriers are mediators of TJA process outcome differences for patients with LEP, the cost differences shown should incentivize investment in interventions to improve communication and care quality for patients with LEP perioperatively and in the postsurgical recovery process. At a time when health care policy is forcing multidisciplinary surgical programs to focus on both cost containment and quality improvement, these findings provide a critical opportunity to enhance efficiencies and quality of care for vulnerable patients with LEP.

## Notes

Supplement. **eAppendix.** Supplemental Methods
