# Supplementary material for: Association of English Language Proficiency With Hospitalization Cost, Length of Stay, Disposition Location, and Readmission Following Total Joint Arthroplasty
Source: JAMA Netw Open. 2022 Mar 10;5(3):e221842. doi: 10.1001/jamanetworkopen.2022.1842 (PMC8914571; doi:10.1001/jamanetworkopen.2022.1842)
Supplement: Supplement. — eAppendix. Supplemental Methods [file jamanetwopen-e221842-s001.pdf]

Supplemental Online Content

Manuel SP, Nguyen K, Karliner LS, Ward DT, Fernandez A. Association of English language proficiency with hospitalization cost, length of stay, disposition location, and readmission following total joint arthroplasty. *JAMA Netw Open*. 2022;5(3):e221842. doi:10.1001/jamanetworkopen.2022.1842

eAppendix. Supplemental Methods

This supplemental material has been provided by the authors to give readers additional information about their work.

© 2022 Manuel SP et al. *JAMA Network Open.*

eAppendix. Supplemental Methods

Reporting of this study follows the Strengthening the Reporting of Observational Studies in Epidemiology (STROBE) guidelines.

Study Design, Population, and Data Collection: This is a cross-sectional cohort study conducted at University of California San Francisco (UCSF) Health, a tertiary care academic medical center that serves a racially and linguistically diverse population. All data for this study was obtained using Clarity, the relational database that stores Epic (Epic Systems Corporation, Verona, Wisconsin) electronic health record (EHR) data in tables. We obtained EHR data for all patients ≥18 years of age who underwent TJA between January 1, 2015 and December 31, 2019 (n=4730). All TJA were performed by orthopedic surgeons at UCSF Health. Patients were excluded if they were deceased at time of disposition (n=9).

Primary outcomes: The primary outcomes of interest included total surgical hospitalization length of stay (time of admission to time of discharge in days), discharge disposition (home versus discharge to other inpatient facility), total cost of hospitalization (direct billing costs in US dollars), and hospital readmission within 30 days from discharge.

Primary predictor: The primary predictor of interest was limited English language proficiency (LEP). Patients were defined as LEP if they met both of the following criteria: (1) documentation of a “preferred language” that was not English, and (2) documentation of request for medical interpreter services. Disparities in criteria 1 and 2 (e.g. preferred language non-English but interpreter services not requested, or preferred language English but interpreter services were requested) was investigated and resolved by manual chart review. In these cases LEP was defined as having a chart note confirming non-English language preference and that an interpreter was used during at least one encounter to assist with communication.

Covariates: Patient demographics collected from Clarity tables included age at admission, self-reported race and ethnicity, self-reported gender, zip code, and primary insurance coverage. For race assignment, patients could decline to answer or self-identify one or more categories from the United States Census list of race categories (White, Black or African American, American Indian or Alaska Native, Asian, and Native Hawaiian or Other Pacific Islander), Other, and Unknown. Options for ethnicity included “Hispanic or Latino” or “Not Hispanic or Latino.” Race and ethnicity were combined into a single five-category variable including White, Asian/Pacific Islander, Black, Hispanic/Latino, and Other. Patients who self-identified as Hispanic or Latino ethnicity were classified as Hispanic/Latino regardless of race. Patients identifying as Native Americans/Alaskan Natives, as well as those with unspecified race or ethnicity, were categorized as Other. Insurance status was categorized as Private, Public, or Medicare.

Data collected from Clarity tables regarding patient preoperative status included American Society of Anesthesiologists physical status classification (as a proxy for severity of illness) and

© 2022 Manuel SP et al. *JAMA Network Open.*

body mass index. Data regarding surgical procedure included elective versus urgent case classification, case length, and estimated blood loss.

Missing Data: There was minimal missing data in our dataset. Where possible, data missing in the initial Clarity download was retrieved via manual chart review. Ten of 4721 (0.2%) observations were missing BMI data points. One observation was missing direct costs data. Two observations were missing discharge disposition data. All other observations had complete data for variables used in the multivariable models. Due to the very small fraction of cases with missing covariates; and the assumption that missingness was completely at random, these missing values were handled using "listwise deletion."

Statistical Analyses: Baseline demographics, patient characteristics, and procedure characteristics were stratified by LEP. Statistical comparisons between LEP versus EP groups were performed using parametric and nonparametric tests as appropriate (χ^2, Fisher's exact, Wilcoxon rank-sum, and *t* tests). Categorical variables were summarised using count/frequency and percentage, and continuous variables were summarised using mean and standard deviations for normally distributed data and median and interquartile ranges for non-normally distributed data.

Next we will fit a series of multivariable regression models to examine the association between LEP and primary outcome variables. The distribution of length of stay and total hospitalization cost both exhibited overdispersion caused by right skew, which was accommodated using negative binomial regression. Logistic regression analyses were used to determine odds ratios for discharge to a skilled facility and 30 day readmission. We first examined the unadjusted association between primary outcomes and LEP alone; and then adjusted for a priori covariates including race/ethnicity, age, gender, primary insurance, ASA status, body mass index, surgical case class, case length, and estimated blood loss. Length of stay and cost of hospitalization incidence rate ratios were additionally adjusted for disposition location. We included variables in models if they were potential confounders regardless of differences in baseline statistics.

All statistical analyses were performed using Stata software version 15.1 (StataCorp LLC, College Station, Texas). All stages of data cleaning and analysis were logged in a Stata version 15.1 do-file by the corresponding author (SPM).

Sensitivity Analyses: Sensitivity analyses investigating the influence of dichotomizing continuous outcome variables using clinically meaningful cut-offs, limiting the multivariable models to variables with significance levels of 0.05 in bivariate analyses, and excluding BMI (the variable with the highest albeit modest level of missingness) from the multivariable models on the study results was also performed. Sensitivity analyses revealed no changes that impacted our study conclusions.

Methodological Limitations: There are some limitations to the current study. First, the quality of our analyses is reliant on the accuracy of the data captured in the EHR. Self-reported demographic data such as primary language may be subject to misclassification. We attempted

© 2022 Manuel SP et al. *JAMA Network Open.*

to minimize this by also including the need for interpreter services within the definition of LEP, and validated the LEP status by manual chart review of 100 patients designated as LEP. Second, our data was limited by what has been recorded into the EHR. Our EHR lacks comprehensive information about patients' health literacy, educational attainment, and social supports. Despite this, we were able to account for many other covariates that might contribute to postoperative recovery after TJA, including age, ASA rating, and BMI. Third, we lacked information on the use of professional interpreters, ad hoc family member interpreters, or languages spoken by staff caregivers. In this study, the presence and utilization of these resources would likely minimize the potential differences we find. Fourth, this study was conducted at a single medical center and generalizability may be limited due to differences in patient and staff demographics. Fifth, there is potential for residual confounding. We attempted to mitigate this by adjusting for as many clinically relevant covariates as possible, including race and ethnicity.

Ethical Review: This study was approved by the University of California, San Francisco Institutional Review Board with waiver of informed consent (protocol 21-34480).

© 2022 Manuel SP et al. *JAMA Network Open.*
